# Body Size at Birth Is Associated with Food and Nutrient Intake in Adulthood

**DOI:** 10.1371/journal.pone.0046139

**Published:** 2012-09-26

**Authors:** Mia-Maria Perälä, Satu Männistö, Niina E. Kaartinen, Eero Kajantie, Clive Osmond, David J. P. Barker, Liisa M. Valsta, Johan G. Eriksson

**Affiliations:** 1 Department of Chronic Disease Prevention, National Institute for Health and Welfare, Helsinki, Finland; 2 Hospital for Children and Adolescents, Helsinki University Central Hospital and University of Helsinki, Helsinki, Finland; 3 MRC Lifecourse Epidemiology Unit, University of Southampton, Southampton General Hospital, Southampton, United Kingdom; 4 Heart Research Center, Oregon Health and Science University, Portland, Oregon, United States of America; 5 Fetal Programming Research Chair, King Saud University, Riyadh, Saudi Arabia; 6 Department of Lifestyle and Participation, National Institute for Health and Welfare, Helsinki, Finland; 7 Data Collection and Exposure, European Food Safety Authority, Parma, Italy; 8 Department of General Practice and Primary Health Care, University of Helsinki, Helsinki, Finland; 9 Unit of General Practice, Helsinki University Central Hospital, Helsinki, Finland; 10 Folkhälsan Research Center, Helsinki, Finland; 11 Vaasa Central Hospital, Vaasa, Finland; The University of Texas M. D. Anderson Cancer Center, United States of America

## Abstract

**Background:**

Small body size at birth is associated with an increased risk of cardiovascular disease and type 2 diabetes. Dietary habits are tightly linked with these disorders, but the association between body size at birth and adult diet has been little studied. We examined the association between body size at birth and intake of foods and macronutrients in adulthood.

**Methodology/Principal Findings:**

We studied 1797 participants, aged 56 to 70, of the Helsinki Birth Cohort Study, whose birth weight and length were recorded. Preterm births were excluded. During a clinical study, diet was assessed with a validated food-frequency questionnaire. A linear regression model adjusted for potential confounders was used to assess the associations. Intake of fruits and berries was 13.26 g (95% confidence interval [CI]: 0.56, 25.96) higher per 1 kg/m^3^ increase in ponderal index (PI) at birth, and 83.16 g (95% CI: 17.76, 148.56) higher per 1 kg higher birth weight. One unit higher PI at birth was associated with 0.14% of energy (E%) lower intake of fat (95% CI: -0.26, -0.03) and 0.18 E% higher intake of carbohydrates (95% CI: 0.04, 0.32) as well as 0.08 E% higher sucrose (95% CI: 0.00, 0.15), 0.05 E% higher fructose (95% CI: 0.01, 0.09), and 0.18 g higher fiber (95% CI: 0.02, 0.34) intake in adulthood. Similar associations were observed between birth weight and macronutrient intake.

**Conclusions:**

Prenatal growth may modify later life food and macronutrient intake. Altered dietary habits could potentially explain an increased risk of chronic disease in individuals born with small body size.

## Introduction

Epidemiological studies have shown that a sub-optimal environment in fetal life may program the development of metabolic diseases including cardiovascular disease [1] and type 2 diabetes [2] in adult life. The possible mechanisms behind the associations between prenatal growth and the development of these diseases in later life are not fully understood. Lifestyle factors such as unhealthy dietary habits and physical inactivity are important not only as potential factors influencing obesity, but they are also independent and modifiable risk factors for cardiovascular disease and type 2 diabetes [3], [4]. Therefore, it has been proposed that one possible mechanism behind the association between a small body size at birth and an increased risk of chronic diseases is early programming of lifestyle factors [5]. Previous meta-analysis supports this hypothesis showing that low birth weight is related to lower physical activity in adulthood [6]. In addition, there is evidence from animal studies that early environment may alter food preferences in later life [7], [8]. However, there are only a few studies that have investigated this in humans. Two previous epidemiological cohorts have observed that famine during gestation was associated with an increased intake of fat in later life [9], [10], while one study of young adults [11] showed that prenatal growth retardation was related to the preference to eat a high carbohydrate diet.

To the best of our knowledge, no information has been published about the association between body size at birth, other than birth weight, and adult life food intake. Therefore, the aim of the present study was to assess whether ponderal index (PI, weight [kg]/length [m^3^]) at birth and birth weight and length is associated with food and macronutrient intake in adult life in a large cohort of men and women born in Helsinki, Finland, between 1934 and 1944.

## Methods

### Ethic Statement

The study was approved by the Ethics Committee of Epidemiology and Public Health of the Hospital District of Helsinki and Uusimaa. Written informed consent was obtained from each participant.

### Design and Study Population

The subjects are all participants in the Helsinki Birth Cohort Study (HBCS) originally consisting of 4630 men and 4130 women. As previously described [12], they were born as singletons at Helsinki University Central Hospital between 1934 and 1944, attended child welfare clinics in the city, and lived in Finland in 1971, when a unique identification number was allocated to each member of the Finnish population. Their birth records included date of birth, weight and length at birth, and last menstrual period of the mother. In order to obtain a sample size of over 2000 individuals for a clinical study, we used random number tables to select 2902 participants living in the greater Helsinki area [13]. Of these, 2003 men (n = 928) and women (n = 1075) attended the clinical examination between August 2001 and March 2004.

The participants attended the clinic after an overnight fast. Height was measured to the nearest 0.1 cm and weight, to 0.1 kg. Body mass index (BMI) was calculated as weight in kilograms divided by the square of height in meters. A team of three trained research nurses performed all measurements. Participants were also asked about their medical history and current medication, using standardized questionnaires at the clinic. In addition, educational attainment and smoking and exercise habits were obtained from a postal questionnaire before the clinical examination [13]. Educational attainment was categorized into three groups according to the number of years in school: basic (≤9 years of education); secondary (10–12 years) and higher (≥13 years of education). The participants were defined as current smokers if they smoked one or more cigarettes per day. We defined those exercising at a level comparable to brisk walking three or more times per week as physically active.

### Dietary Assessment

Diet was assessed by a validated, self-administered, 128-item, food-frequency questionnaire (FFQ) [14], [15]. The FFQ was designed to assess the usual diet over the previous 12 months. The participants were asked to indicate the average intake frequency of each food-item and mixed dish presented as 12 subgroups, for example, dairy products and vegetables. Response options for the nine possible frequency categories ranged from never or seldom to six or more times a day. The portion sizes for each food item were specified in natural units (e.g., one banana, 190 g), common household measures (e.g., one glass of milk, 170 g), or portions (e.g., one portion of meat soup, 300 g). Portion sizes were based on the national Findiet Survey and present the most commonly used portion sizes in Finland.

At the clinic, participants completed the FFQ, which was then checked by a research nurse. The food intake data were entered and processed at the National Institute for Health and Welfare, Finland with the in-house calculation software Finessi utilizing the National Food Composition Database, FINELI ® [16]. The daily food intake was calculated by multiplying the frequency of food consumption by fixed portion sizes to obtain the weight of each listed food-item consumed as an average per day. The average daily intake of nutrients was calculated by multiplying the gram intake per day of each food by its nutrient content. Dietary glycemic index (GI) and glycemic load (GL) were calculated by using the GI -database [17]. Dietary GI was calculated as the weighed mean of the GI values of the carbohydrate-containing foods, where weighting is based on the proportion of the total carbohydrate content provided by each food. Dietary GL was calculated by multiplying the dietary GI value with the carbohydrate content of the diet and dividing by 100. For the purpose of the present study, food items and mixed dishes were combined into 15 food groups on the basis of culinary use, nutrient profile, and nutritional relevance. At first, all food items and mixed dishes were broken down into simple ingredients (e.g. wheat, milk, and ice cream), which were then classified into their appropriate food groups.

### Statistical Analysis

Participants were excluded if their FFQ was incomplete (n = 2) or if their calculated energy intake was under 2.7 MJ/d or over 25.5 MJ/d, corresponding to 0.5% at both ends of the self-reported daily energy intake distributions for men and women (n = 20). In all, 180 participants were excluded because their gestational age at birth was under 37 completed weeks, over 44 completed weeks, or was not recorded. In addition, BMI was not recorded for two subjects and for one, it was considered too high to be included in the analysis (68.4 kg/m^2^). Furthermore, one participant was excluded because his fruit and berry intake was over 5 kg/d. The final analysis comprised 1797 participants.

Intake of foods and macronutrients were adjusted for energy intake by calculating the proportion of energy (E%) or by using the residual method (fiber, dietary GL, and food groups) [18]. The relationship between body size at birth and food and macronutrient intake was examined by linear regression analysis. There was no interaction between the effects of sex and PI at birth or birth weight or length on food and macronutrient intake, and therefore, pooled analyses are presented. Models were adjusted for potential confounding variables, which included sex and current age (Model 1), and Model 2 was further adjusted for current BMI, smoking, education, and gestational age. Additional analyses were also adjusted for physical activity.

Results are expressed as mean (standard deviation [SD]) or regression coefficients (95% confidence intervals [CI]). All statistical analyses were done using the PASW Statistics version 18 for Windows® (SPSS Inc., Chicago, IL, USA); the level of significance was P<0.05.

## Results

### Participants’ Characteristic

The final analysis included 836 men and 961 women. The basic characteristics and nutrient and food intake of the participants are described in **Table 1**. Men were born with a higher birth weight and were heavier in adult life compared with women; however, the mean PI at birth and BMI in adult life were similar in men and women. The intake of fat and protein were similar in men and women; however, men had a higher intake of alcohol and lower intake of carbohydrate compared with women. In addition, dietary GI was similar in men and women although men had higher dietary GL compared with women.

**Table 1:** Birth measurements and adult clinical data^1^.

		MEN	WOMEN
n		836	961
Birth data			
	Weight (g)	3514 (464)	3381 (438)
	Gestational age (wk)	40.05 (1.42)	40.14 (1.44)
	Length (cm)	50.8 (1.9)	50.1 (1.7)
	Ponderal index (kg/m^3^)	26.7 (2.3)	26.8 (2.2)
Adult data			
	Age (y)	61.5 (2.8)	61.5 (3.1)
	Weight (kg)	85.9 (13.6)	74.0 (13.8)
	Height (cm)	176.8 (6.0)	163.3 (5.7)
	Body mass index (kg/m^2^)	27.5 (4.0)	27.8 (5.0)
	Educational attainment 2		
	Basic (%)	39.5	43.7
	Secondary (%)	24.0	22.9
	Higher (%)	36.4	33.4
	Smoker (%) 3	21.9	15.9
	Physically active (%) 4	46.1	42.6
Nutrient intake			
	Energy intake (MJ)	10.34 (3.63)	8.56 (2.96)
	Carbohydrate (E%)	45.5 (6.7)	47.7 (6.6)
	Fat (E%)	33.7 (5.7)	33.1 (5.3)
	Protein (E%)	16.6 (2.5)	17.4 (2.5)
	Alcohol (E%)	4.4 (5.2)	1.9 (2.7)
	Dietary GI	66 (5)	62 (4)
	Dietary GL	180 (68)	146 (54)
Food intake			
	Cereals (g)	172.5 (82.8)	152.3 (68.4)
	Fruits and berries (g)	397.4 (325.3)	446.1 (328.5)
	Vegetables and roots (g)	238.0 (150.6)	319.9 (214.8)
	Potato and potato products (g)	163.2 (116.0)	106.6 (73.2)
	Fish (g)	62.5 (50.7)	47.0 (47.8)
	Meat (g)	191.4 (127.7)	136.4 (88.4)
	Milk and milk products (g)	512.4 (372.9)	512.6 (329.6)
	Fats (g)	43.3 (19.9)	38.1 (18.6)
	Sugar and confectionery (g)	32.1 (32.3)	25.8 (24.2)

1 Results are expressed as mean (SD) or proportions.

2 Educational attainment; three categories by approximate years studied (0–9 = basic, 10–12 = secondary, 13 or more = higher).

3 Smoking one or more cigarettes per day.

4 Proportion of people who exercised 3 or more times per week.

### Body Size at Birth and Food Intake

There was an association between body size at birth and food intake in adulthood, such that each unit increase in PI at birth was associated with 13.26 g (95% CI: 0.56, 25.96) higher intake of fruits and berries, and a 1 kg increase in birth weight was related to 83.16 g (95% CI: 17.76, 148.56) higher intake of fruits and berries (**Table 2**). PI at birth and birth weight was also inversely associated with intake of potato and potato products; however, associations did not reach the statistically significant level. In addition, higher PI at birth predicted higher intake of rye and rye products in adulthood. No other statistically significant associations were observed between PI at birth or birth weight and food intake in adulthood. Birth length did not associate significantly with food intake (data not shown). Adjusting food intake for physical activity did not attenuate the results (data not shown).

**Table 2:** The association between ponderal index (PI) at birth and birth weight and energy adjusted food intake in adulthood.

	PI at birth (kg/m^3^)				Birth weight (kg)			
Food group (g)	Model 1		Model 2		Model 1		Model 2	
	Regression coefficient(95% CI)	P	Regression coefficient(95% CI)	P	Regression coefficient(95% CI)	P	Regression coefficient(95% CI)	P
Cereals	1.44 (−1.10, 3.97)	0.27	1.77 (−0.80, 4.33)	0.18	5.62 (−6.78, 18.03)	0.37	6.51 (−6.70, 19.71)	0.33
Rye and rye products	1.41 (0.06, 2.76)	0.041	1.54 (0.16, 2.91)	0.028	6.43 (−0.18, 13.03)	0.057	6.77 (−0.31, 13.84)	0.061
Wheat and wheat products	−0.09 (−1.80, 1.62)	0.92	−0.21 (−1.94, 1.53)	0.81	0.60 (−7.78, 8.98)	0.89	−0.22 (−9.18, 8.73)	0.96
Fruits and berries	10.90 (−1.93, 23.74)	0.096	13.26 (0.56, 25.96)	0.041	68.98 (6.18, 131.78)	0.031	83.16 (17.76, 148.56)	0.013
Vegetables and roots	2.75 (−4.86, 10.36)	0.48	2.62 (−5.10, 10.35)	0.51	2.45 (−34.80, 39.70)	0.90	−4.53 (−44.31, 35.24)	0.82
Potato and potato products	−3.02 (−6.73, 0.69)	0.11	−3.49 (−7.17, 0.19)	0.063	−11.28 (−29.46, 6.90)	0.22	−16.53 (−35.51, 2.45)	0.088
Fish and fish products	−1.27 (−3.19, 0.65)	0.19	−1.37 (−3.33, 0.60)	0.17	−2.28 (−11.90, 7.34)	0.64	−3.22 (−13.59, 7.15)	0.54
Total meat	0.09 (−3.81, 3.99)	0.96	−1.51 (−5.34, 2.32)	0.44	5.10 (−13.97, 24.16)	0.60	−8.14 (−27.84, 11.56)	0.42
Red meat	0.69 (−2.82, 4.19)	0.70	−1.01 (−4.45, 2.42)	0.56	−1.82 (−15.32, 18.96)	0.84	−9.93 (−27.60, 7.74)	0.27
Processed meat	−0.07 (−2.16, 2.02)	0.95	−1.13 (−3.03, 0.78)	0.25	−1.00 (−11.20, 9.20)	0.85	−8.30 (−18.11, 1.52)	0.097
Milk and milk products	5.57 (−7.80, 18.95)	0.41	5.08 (−8.57, 18.72)	0.47	10.47 (−55.38, 76.32)	0.76	19.14 (−51.55, 89.83)	0.60
Fats	0.24 (−0.41, 0.90)	0.47	0.20 (−0.46, 0.86)	0.55	0.85 (−2.37, 4.07)	0.61	0.43 (−2.99, 3.86)	0.80
Butter and butter spread	0.08 (−0.46, 0.61)	0.78	0.06 (−0.48, 0.60)	0.83	0.51 (−2.09, 3.11)	0.70	0.62 (−2.14, 3.38)	0.66
Margarine and fat spread	0.21 (−0.15, 0.56)	0.25	0.20 (−0.17, 0.56)	0.29	0.01 (−1.73, 1.75)	0.99	0.12 (−1.75, 1.99)	0.90
Sugar and confectionery	0.12 (−0.97, 1.21)	0.83	0.27 (−0.84, 1.38)	0.63	3.29 (−2.03, 8.61)	0.23	4.89 (−0.81, 10.60)	0.093

Mean difference (95% CI) in daily food intake is given to the increase of 1 kg/m^3^ in PI at birth or 1 kg in birth weight (n = 1797).

Model 1: Adjusted for sex and current age, tested by linear regression model.

Model 2: Adjusted for sex, current age and BMI, education, smoking, and gestational age, tested by linear regression model.

### Body Size at Birth and Macronutrient Intake

Body size at birth was associated with the macronutrient intake in later life in such a way that a 1 kg/m^3^ higher PI at birth was associated with 0.14 E% (95% CI: −0.26, −0.03) lower intake of total fat, and a 1 kg increase in birth weight was associated with 0.62 E% (95% CI: −1.21, −0.03) lower intake of total fat (**Table 3**). PI at birth and birth weight was also inversely associated with intake of monounsaturated fatty acids. No such associations were observed between body size at birth and saturated fatty acids or polyunsaturated fatty acids. PI at birth as well as birth weight was positively associated with intake of total sugars as well as fructose and sucrose. A one-unit increase in PI at birth was also associated with 0.18 E% (95% CI: 0.04, 0.32) higher intake of carbohydrates and 0.18 g (95% CI: 0.02, 0.34) higher intake of fiber. In addition, with adjustment for sex and current age, higher birth weight was associated with lower dietary GI; however, the association became statistically non-significant after adjustment for current BMI, education, smoking, and gestational age. Birth length was not significantly associated with macronutrient intake or dietary GI or GL in adulthood (data not shown). Adjusting the macronutrient intake for physical activity did not attenuate the results (data not shown).

**Table 3:** The association between ponderal index (PI) at birth and birth weight and nutrient intake in adulthood.

	PI at birth (kg/m^3^)				Birth weight (kg)			
Dietary intakes	Model 1		Model 2		Model 1		Model 2	
	Regression coefficient(95% CI)	P	Regression coefficient(95% CI)	P	Regression coefficient(95% CI)	P	Regression coefficient(95% CI)	P
Energy (kJ)	27.9 (−41.1, 97.0)	0.43	22.0 (−48.2, 92.2)	0.54	247.7 (−90.7, 586.2)	0.15	221.0 (−140.6, 582.6)	0.23
Carbohydrate (E%)	0.14 (−0.01, 0.28)	0.055	0.18 (0.04, 0.32)	0.010	0.36 (−0.32, 1.04)	0.30	0.57 (−0.15, 1.29)	0.12
Sugars (E%)	0.13 (−0.01, 0.26)	0.054	0.16 (0.03, 0.29)	0.015	0.57 (−0.07, 1.20)	0.079	0.82 (0.15, 1.49)	0.016
Fructose (E%)	0.04 (0.00, 0.09)	0.051	0.05 (0.01, 0.09)	0.019	0.24 (0.03, 0.45)	0.029	0.27 (0.05, 0.49)	0.017
Sucrose (E%)	0.05 (−0.02, 0.13)	0.18	0.08 (0.00, 0.15)	0.050	0.29 (−0.09, 0.66)	0.13	0.44 (0.04, 0.83)	0.030
Fiber (g)^1^	0.15 (−0.02, 0.32)	0.074	0.18 (0.02, 0.34)	0.031	0.65 (−0.16, 1.46)	0.12	0.78 (−0.06, 1.63)	0.070
Dietary GI	−0.08 (−0.18, 0.01)	0.093	−0.08 (−0.18, 0.02)	0.11	−0.51 (−0.99, −0.03)	0.036	−0.49 (−0.99, 0.02)	0.060
Dietary GL^1^	0.21 (−0.25, 0.67)	0.37	0.35 (−0.11, 0.81)	0.13	−0.22 (−2.45, 2.02)	0.85	0.41 (−1.95, 2.77)	0.73
Protein (E%)	−0.01 (−0.06, 0.04)	0.67	−0.03 (−0.08, 0.03)	0.30	−0.01 (−0.27, 0.25)	0.96	−0.14 (−0.41, 0.14)	0.33
Fat (E%)	−0.11 (−0.22, 0.01)	0.061	−0.14 (−0.26, −0.03)	0.013	−0.39 (−0.95, 0.17)	0.18	−0.62 (−1.21, −0.03)	0.039
SFA (E%)	−0.03 (−0.08, 0.03)	0.35	−0.04 (−0.09, 0.02)	0.20	−0.15 (−0.42, 0.12)	0.27	−0.15 (−0.43, 0.13)	0.30
MUFA (E%)	−0.04 (−0.09, 0.01)	0.076	−0.06 (−0.10, −0.01)	0.015	−0.11 (−0.33, 0.12)	0.35	−0.24 (−0.48, −0.01)	0.039
PUFA (E%)	−0.02 (−0.04, 0.01)	0.22	−0.02 (−0.05, 0.01)	0.11	−0.03 (−0.16, 0.10)	0.63	−0.09 (−0.22, 0.04)	0.19
Alcohol (E%)	−0.01 (−0.10, 0.07)	0.76	−0.01 (−0.09, 0.08)	0.85	0.02 (−0.39, 0.44)	0.91	0.17 (−0.27, 0.61)	0.46

Mean difference (95% CI) in daily nutrient intake is given to the increase of 1 kg/m^3^ in PI at birth or 1 kg in birth weight (*n* = 1797).

Abbreviations: E%, per cent of total energy intake; GI, glycemic index; GL, glycemic load; SFA, saturated fatty acids; MUFA, monounsaturated fatty acids; PUFA, polyunsaturated fatty acids.

Model 1: Adjusted for sex and current age, tested by linear regression model.

Model 2: Adjusted for sex, current age and BMI, education, smoking, and gestational age, tested by linear regression model.

1 Adjusted for energy by residual method.

## Discussion

We showed that small body size at birth was associated with lower consumption of fruits and berries and rye and rye products in Finnish men and women aged 56 to 70. In addition, those who were small at birth had higher intake of fat and lower intake of carbohydrates as well as sucrose, fructose, and fiber. We observed that a 1 kg higher birth weight was associated with about 83 g higher daily intake of fruits and berries; thus, weekly consumption of fruits and berries was over 580 g higher. Lower consumption of fruits and berries reflects an unhealthy diet, which may increase the risk of cardiovascular disease [19]. Indeed, the importance of low fruit and berry intake as well as vegetable intake is highlighted by WHO [20], ranking it 6^th^ as a risk factor of death world-wide, with a higher proportion of attributable deaths than, for example, overweight or physical inactivity. Therefore, our results suggest that intrauterine growth may modify food intake in adult life, which may subsequently affect health outcomes in later life.

It has been proposed that conditions during the fetal period may alter dietary habits in later life. Animal models support this hypothesis by showing that rats whose mothers were fed a low-protein diet during gestation had a preference for a high-fat diet and an aversion to a high-carbohydrate diet [8]; however, another animal study showed no effect of birth weight on later food preferences [7]. There is also evidence from studies on young children that fat intake increased with decreasing birth weight [21], [22]. To our knowledge, only a few epidemiological studies have examined whether body size at birth is associated with macronutrient intake in adult life and only one has focused on body size at birth and food intake. Lussana and co-workers observed that prenatal exposure to the Dutch famine in early gestation was associated with a preference for greater fat intake in later life [9]. They did not, however, find any relationships between birth weight and macronutrient intake. In addition, another study group observed that prenatal exposure to the Dutch famine was related to greater intake of fat compared with sibling controls not exposed to famine [10]. Contrary to our study, they did not investigate whether body size at birth is associated with macronutrient intake. Moreover, Barbieri et al. [11] detected increased intake of carbohydrates in young adult Brazilian women who were born with severe intrauterine growth restriction (IUGR). Although they observed differences in macronutrient intake, IUGR had no effect on the consumption of milk, meat, bread, vegetables, fruits, and fiber nor the total energy derived from sweet foods. Interestingly, in a study of young Finn adults, it was observed that those who were born preterm at below 1500 g had similar macronutrient intake than controls, although they consumed less fruits, berries, vegetables, and milk products (unpublished results). Unlike previous studies that specifically examined the association between famine or intrauterine growth restriction and adult life nutrient intake, we assessed the association between PI at birth as well as birth weight and length and nutrient and food intake. Indeed, we found stronger associations between PI at birth than between birth weight and adult life macronutrient intake. Moreover, in both Dutch famine studies, the fat intake of the study populations was much greater (36 E%) and carbohydrate intake much lower (42 E%) compared with our study. These differences may at least partly explain inconsistent findings between the studies. In addition, it has been proposed that aging may alter food intake and food preferences, which could explain different findings between studies of young and older adults. One recently published study support this finding that in young girls, IUGR contributed to impulsive eating, which may promote increased fat consumption [23]. In accordance with this finding, IUGR is also associated with the consumption of palatable foods in preterm infants [24]. Thus, IUGR may directly program obesigenic eating behaviors in young children, whereas in later life, metabolic changes that are related to prenatal growth may secondarily influence food choices.

There are some potential underlying mechanisms explaining our results. Based on animal studies, macronutrient selection behavior is controlled by the actions of neuropeptides in specific centers of the hypothalamus [25]. It has been proposed that a low-protein diet during gestation may alter the expression of these peptides, such as neuropeptide Y and galanin [26], [27]. However, whether these peptides play a role in macronutrient selection behaviors in humans as well is still unknown. It has also been shown in an animal model [28] as well as in humans [29] that maternal diet during pregnancy may influence postnatal preference for the same diet. In addition, leptin is also related to taste perception [30]. It has been shown that subjects who have elevated leptin levels need a higher concentration of sweeteners, such as fructose to detect the sweet stimulus, than subjects with lower leptin levels [31], [32]. Therefore, blunting sweet taste may lead to a reduced consumption of sweet foods [33]. Increased leptin secretion has been observed among participants who were born with low birth weight [34], [35]. Thus, altered leptin metabolism could potentially be involved in the food consumption such as the decreased intake of sweet fruits and berries, in participants who have experienced retarded growth during prenatal life.

The main strength of the current study was the use of a large cohort consisting of both men and women. In addition, birth data were obtained from reliable records and not based on merely recalled values. A further strength of the study was the use of a validated FFQ, which measures the whole diet and which was found to rank participants reasonably well according to their food and nutrient intake [14], [15]. We have already discussed the limitations of the HBCS elsewhere [36], [37]. The participation rate of the clinical examination was 69% of those invited. Our results were, however, based on internal comparisons within the sample. Selection bias would be expected to affect the results only if the association between prenatal growth and adult dietary intake was different in participants compared with non-participants. This is unlikely but cannot be excluded. In addition, we acknowledge that our study may have some potential limitations related to the use of the FFQ because participants may overestimate consumption of food considered as healthy and underreport intake of food considered as unhealthy [14]. While our results survived adjustment for socioeconomic status as measured by educational attainment, residual confounding remains a possibility with regard to items such as fruits and berries. However, fruits and berries are relatively inexpensive and available round the year in Finland. Finally, we do not have the data of childhood food intake, therefore, our results are cross-sectional and do not allow conclusions on causality.

In conclusion, the association between a small body size at birth and lower intake of carbohydrates and especially fruits and berries and higher intake of fats suggest that adult dietary habits might be, in part, programmed during prenatal life. Therefore, dietary counseling could be especially beneficial for those born with a small body size as they have an increased risk of developing chronic disease in later life. However, further studies are needed to confirm our observations on other populations and in different ethnic groups.
